# Ferulic Acid Mitigates Growth and Invasion of Esophageal Squamous Cell Carcinoma through Inducing Ferroptotic Cell Death

**DOI:** 10.1155/2022/4607966

**Published:** 2022-10-11

**Authors:** Yu Cao, Hong Zhang, Jianming Tang, Rui Wang

**Affiliations:** Department of Thoracic Surgery, The Third Affiliated Hospital of Chongqing Medical University, Chongqing 401120, China

## Abstract

**Objective:**

Ferroptosis is an iron- and ROS-dependent form of cell death initiated by lipid peroxidation. The rapidly developing study of ferroptosis has facilitated its application in cancer therapeutics. The current study is aimed at investigating the functional property of ferulic acid (FA, a phenolic acid substance) on inducing ferroptosis in antiesophageal squamous cell carcinoma (ESCC).

**Methods:**

ESCC cells were administrated with gradient doses of FA or with ferroptosis inhibitor deferoxamine. Cellular growth was measured with CCK-8 and colony formation experiments. LDH, caspase-3, MDA, SOD, GSH, and iron were assayed with corresponding kits. Apoptotic level was evaluated through Annexin V-FITC apoptosis staining, with migration and invasion utilizing Transwell assays. Through quantitative RT-PCR, angiogenesis-relevant genes VEGFA and PDGFB were detected. ROS generation was measured via DCFH-DA probe. Immunoblotting was conducted for monitoring ACSL4, SLC7A11, HO-1, and GPX4.

**Results:**

FA administration observably mitigated cellular viability and colony formation capacity and motivated LDH release, caspase-3 activity, and apoptosis in EC-1 and TE-4 cells. In addition, migration and invasion together with angiogenesis of ESCC cells were restraint by FA. FA exposure led to the increase of MDA content, ROS production, and iron load as well as the reduction of SOD activity and GSH content. Also, FA augmented the activities of ACSL4 and HO-1, with lessening SLC7A11 and GPX4. Nonetheless, deferoxamine restrained the effect of FA on ESCC ferroptosis.

**Conclusion:**

Altogether, FA may act as a ferroptosis inducer and thus attenuates cell growth and invasion of ESCC, which boosts the clinical application of FA in ESCC therapeutics.

## 1. Introduction

Esophageal cancer remains one of the most lethal cancers worldwide [1]. There were an estimated 604,100 newly diagnosed cases and 544,100 death cases globally in 2020 [2]. 957,000 new cases and 880,000 deaths are expected by 2040. Males are predominant in esophageal cancer, with male-to-female incidence and mortality ratios of 2 : 1 and 3 : 1 [3]. Esophageal squamous cell carcinoma (ESCC) is the dominating histological form, occupying 85% of all esophageal cancer cases, especially African and Asian populations [4]. Risk factors for ESCC mainly cover genetics, diet and nutrition, gastric atrophy, infection and microbiology, metabolism, epidemiology, and environment, etc. [5]. The progression of ESCC primarily contains pure epithelial hyperplasia, dysplasia, preinvasive carcinoma, invasive carcinoma, and metastasis stages [6]. Surgical resection and chemoradiotherapy remain the prime therapeutic options for combating this deadly malignancy [7]. Nonetheless, clinical outcomes are still unfavorable as a result of the limited efficacy together with serious adverse reactions. Despite the development of large-scale next-generation sequencing, the development of molecularly targeted agents (cetuximab, bevacizumab, agents targeting surface antigens and immune checkpoints, etc.) is still in its early stages. The new agents may be applied alone or combined with other therapeutic options, thus improving the therapeutic efficacy together with outcomes [8–10]. Nonetheless, adverse events, first-rank doses, and available combination regimens still require in-depth exploration.

Ferroptosis is a reactive oxygen species- (ROS-) dependent form of cell death with two major biochemical traits, iron accumulation and lipid peroxidation [11]. Ferroptotic cells often exhibit necrotic-like morphological alterations such as disruption of plasma membrane integrity, swelling of cytoplasm and cytoplasmic organelles, and moderate condensation of chromatins [12]. At the ultrastructural levels, cells that experience ferroptosis often display mitochondrial abnormalities. Ferroptotic cell death occurs primarily by extrinsic or transporter-dependent signaling (reduced cysteine or glutamine uptake, enhanced iron uptake, etc.) as well as intrinsic or enzyme-mediated signaling (GPX4 suppression, etc.) [13]. Excessive or defective ferroptotic cell death may result in pathological cell damage and malignant processes. Accumulated evidence demonstrates the potential of inducing ferroptosis for ESCC treatment. For instance, SLC7A11-mediated suppression of ferroptotic cell death induces NRF2-associated the resistance of ESCC radiotherapy [14]. ESCC stem-like cells hit back ferroptosis through activating Hsp27-GPX4 signaling [15]. 5-Aminolevulinic acid exerts an anti-ESCC property through inducing ferroptosis [16]. Oridonin enhances ferroptosis of ESCC cells via attenuating gamma-glutamyl cycle [17]. circPVT1 mitigates 5-fluorouracil chemosensitivity via resisting ferroptosis in ESCC cells [18]. Hence, more efforts are required for the design and development of anti-ESCC agents on the basis of ferroptosis induction.

Ferulic acid (4-hydroxy-3-methoxycinnamic acid, FA), a phenolic acid substance, is broadly distributed in the plant kingdom, which is usually covalently conjugated with lignin, polysaccharide, etc. of plant cell walls [19]. As a bioactive substance with multiple functions, FA is capable of removing redundant ROS and free radicals, thus resisting oxidative injury as well as lowering inflammatory response [20]. In addition, it exerts a potent anti-cancer property through modulating multiple pathways [21]. Limited evidence proposes the relationships of FA with ferroptosis in pathological state [22]. Nonetheless, the anti-ESCC property and mechanisms of FA are still unexplored. The current study put forward the hypothesis that FA mitigated growth of ESCC via inducing ferroptotic cell death.

## 2. Materials and Methods

### 2.1. Cell Culture

ESCC cells TE-4 and EC-1 (Cell Bank of Shanghai Institute of Cell Biology, China) were cultivated in Roswell Park Memorial Institute (RPMI) 1640 medium (Corning, USA) plus 10% fetal bovine serum (FBS; Invitrogen, USA) together with 1% penicillin/streptomycin (HyClone, USA) at 37°C in 5% CO_2_. FA (purity ≥ 95%) was acquired from Aladdin (China). ESCC cells were administrated with a gradient of FA dissolved by dimethyl sulfoxide (DMSO) for distinct time points. Additionally, ESCC cells were exposed to 50 *μ*M deferoxamine (DFO; Aladdin, China) to mitigate ferroptosis.

### 2.2. Cell Viability Assay

ESCC cells were seeded onto 96-well plates (5 × 10^3^ cells/well), and cell viability was assayed with Cell Counting Kit-8 (CCK-8; Abcam, USA) in line with the manufacturer's protocol. Viable cells were quantified through detecting the absorbance value at 450 nm utilizing a microplate reader.

### 2.3. Lactate Dehydrogenase (LDH) Release and Caspase-3 Activity Assay

LDH release level was monitored with LDH cytotoxicity assay kit (Abcam, USA) in line with the manufacturer's instruction. In brief, ESCC cells were lysed and exposed to pyruvate and nicotinamide adenine dinucleotide (NADH) for 15 min at 37°C. Absorbance value at 530 nm was quantified with a microplate reader. Caspase-3 activity was measured utilizing its assay kit (Abcam, USA) in accordance with the manufacturer's protocols. Absorbance value at 405 nm was quantified with a microplate reader.

### 2.4. Colony Formation Assay

ESCC cells were seeded onto a 6-well plate (1 × 10^3^ cells/well) and inculcated for 2 weeks. The colonies were fixed with 4% paraformaldehyde for 15 min and dyed with 0.5% crystal violet for 5 min. Afterwards, the colonies were counted under a light microscope.

### 2.5. Annexin V-FITC Apoptosis Staining

Apoptotic level was assayed with Annexin V-FITC/propidium iodide (PI) apoptosis detection kit (Beyotime, China). ESCC cells were collected via trypsinization without EDTA. After washing twice with ice-cold phosphate buffer saline (PBS), they were prepared as a single-cell suspension and dual-dyed utilizing 5 *μ*L Annexin V-FITC together with 10 *μ*L propidium iodide away from light for 5 min at room temperature (RT). The proportion of apoptotic cells was quantified with FACSCalibur flow cytometer (BD Biosciences, USA).

### 2.6. Transwell Assays

Transwell assays were adopted for detecting cellular migration and invasion. ESCC cells were cultivated in serum-free medium (SFM) for 24 h, which were inoculated into the upper chamber coated with Matrigel (for invasion assay) or uncoated (for migration assay). 200 *μ*L SFM with 1 × 10^5^ ESCC cells was added to the upper chamber, with the same medium plus 10% FBS adding to the lower chamber. Afterwards, the cells were grown at 37°C for 24 h. The migrated or invaded cells attached to the lower surface of the upper chamber were fixed with 4% paraformaldehyde, and dyed with 0.1% crystal violet. Images were photographed and counted.

### 2.7. RNA Extraction and Quantitative RT-PCR Analysis

Total RNA of ESCC cells was extracted with TRIzol reagent. RNA concentration and purity were assessed utilizing spectrophotometry. Complementary DNA was synthesized with PrimeScript RT Reagent kit (Takara, Japan). RT-PCR was conducted on CFX96 PCR system (Bio-Rad, USA) together with SYBR Green Supermix (Bio-Rad). Primer sequences included: VEGFA, 5′-AGGGCAGAATCATCACGAAGT-3′ (forward), 5′-AGGGTCTCGATTGGATGGCA-3′ (reverse); PDGFB, 5′-CTCGATCCGCTCCTTTGATGA-3′ (forward), 5′-CGTTGGTGCGGTCTATGAG-3′ (reverse); and GAPDH, 5′-CGAGATCCCTCCAAAATCAA-3′ (forward), 5′-TGTGGTCATGAGTCCTTCCA-3′ (reverse). Gene expression was computed with 2^-*ΔΔ*Ct^ approach.

### 2.8. Measurement of Malondialdehyde (MDA), Superoxide Dismutase (SOD), and Glutathione (GSH)

In line with the manufacturer's instruction, MDA content and SOD activity were monitored and normalized in ESCC cells that were collected and ultrasonicated through corresponding kits (Abcam, USA). Total quantity of glutathione was tested by GSH and GSSG assay kit following the manufacturer's instruction (Abcam, USA). GSH content was assessed in contrast to the standard curves of GSH.

### 2.9. ROS Generation Assay

ESCC cells were seeded onto 96-well plates and exposed to 10 *μ*M dichlorodihydrofluorescein diacetate (DCFH-DA; Sigma-Aldrich, USA) probe for 20 min at 37°C. Thereafter, they were instantly submitted to a fluorescence microscope (Olympus, Tokyo, Japan).

### 2.10. Iron Detection

Iron content in ESCC cells was monitored with iron assay kit in line with the manufacturer's instruction (Beyotime, China). Cells were homogenized via five volumes of iron assay buffer and centrifugated at 13,000 g lasting 10 min at 4°C. Thereafter, iron reducer was added to supernatant mixture and treated for 30 min at room temperature, followed by iron probe protecting against light for 1 h. The absorbance value was monitored at 593 nm.

### 2.11. Immunoblotting

Protein extraction was conducted through homogenizing ESCC cells in radioimmunoprecipitation assay (RIPA) reagent (Sigma-Aldrich, USA) supplemented with protease inhibitors. The homogenate was centrifugated at 12,000 rpm at 4°C for 20 min, and the supernatant was harvested. The concentration of proteins was assessed with bicinchoninic acid (BCA) protein assay kit (Pierce, USA). Afterwards, the proteins were loaded onto SDS polyacrylamide gel, separated through electrophoresis, and transferred onto nitrocellulose membranes via electroblotting. The membranes were blocked by 5% skim milk for 1 h at RT, followed by incubation with primary antibody of ACSL4 (1/10000; ab155282; Abcam), SLC7A11 (1/1000; ab175186), HO-1 (1/2000; ab52947), GPX4 (1/2000; ab41787), or GAPDH (1/10000; ab128915) diluted in 5% skim milk overnight at 4°C. After washing with PBS, the membranes were incubated with horseradish peroxidase- (HRP-) conjugated anti-IgG antibody (1/500; ab7085) for 1 h at RT. Signal was visualized with enhanced chemiluminescence (ECL) and viewed utilizing FluorChem® M MultiFluor system (Cell Biosciences, USA).

### 2.12. Statistics and Analysis

All data are expressed as the standard error of mean. One- or two-way analysis of variance (ANOVA) test was conducted with GraphPad Prism 8.0.1 software (GraphPad, USA) for comparing differences between groups. *p* < 0.05 denotes statistical significance.

## 3. Results

### 3.1. FA Exposure Mitigates Cellular Viability and Induces LDH Release and Caspase-3 Activity of ESCC Cells

**Figure 1:**
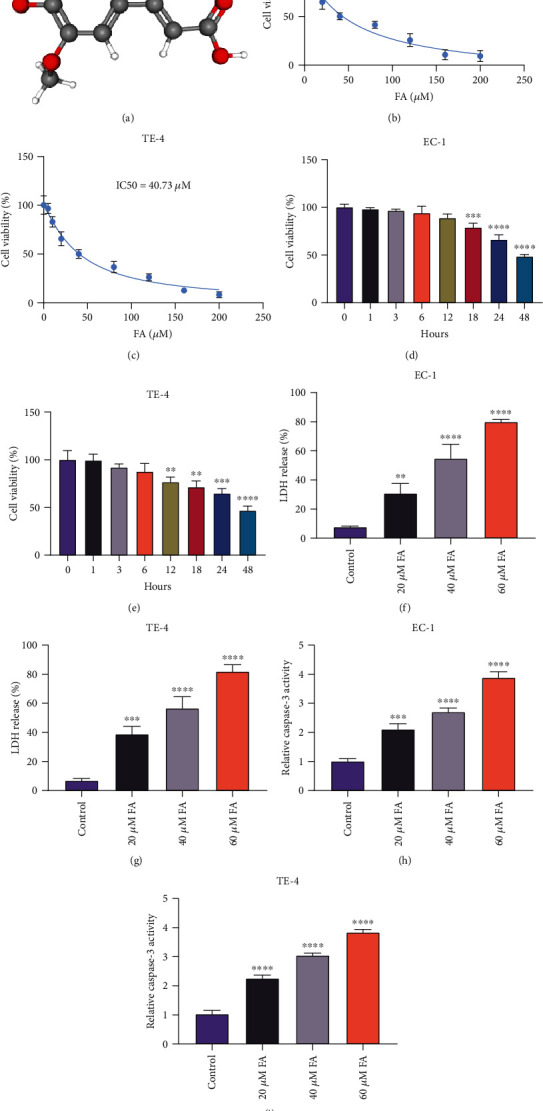
FA exposure mitigates cellular viability and induces LDH release and caspase-3 activity of ESCC cells. (a) Chemical structure of FA from the PubChem database. (b, c) Viable EC-1 and TE-4 cells that were exposed to gradient doses of FA for 48 h were quantified through CCK-8, and IC50 values of FA were computed. (d, e) 40 *μ*M FA was administrated to EC-1 and TE-4 cells for a series of time points, and cellular viability was monitored via CCK-8. (f, g) LDH release level was monitored in EC-1 and TE-4 cells with 48 h administration of 20 *μ*M, 40 *μ*M, and 60 *μ*M FA through LDH cytotoxicity assay kit. (h, i) Caspase-3 activity was quantified in EC-1 and TE-4 cells with 48 h exposure of 20 *μ*M, 40 *μ*M, and 60 *μ*M FA utilizing caspase-3 activity assay kit. *p* was computed through one- or two-way ANOVA test. Significance level was denoted as ^∗∗^*p* < 0.01, ^∗∗∗^*p* < 0.001, and ^∗∗∗∗^*p* < 0.0001.

Figure 1(a) illustrates the chemical structure of FA that was acquired from the PubChem database. For investigating the property of FA on ESCC cellular viability, gradient doses of FA were administrated to ESCC cells for 48 h. Following 48 h exposure, IC50 values of EC-1 and TE-4 were separately 40.98 *μ*M and 40.73 *μ*M (Figures 1(b) and 1(c)). In addition, the optimal time point was determined when ESCC cells were exposed to 40 *μ*M FA. As illustrated in Figures 1(d) and 1(e), the cellular viability was notably attenuated under 48 h exposure. We also investigated the influence of FA on cytotoxicity of ESCC cells through monitoring LDH release. As a result, 20 *μ*M, 40 *μ*M, and 60 *μ*M FA exposure observably motivated LDH release in EC-1 and TE-4 cells in a dose-dependent manner (Figures 1(f) and 1(g)). In addition, caspase-3 activity was tested in ESCC cells administrated with 20 *μ*M, 40 *μ*M, and 60 *μ*M FA for 48 h. The data showed the notable stimulative function of FA on caspase-3 activity of ESCC cells (Figures 1(h) and 1(i)). Altogether, FA administration led to the reduction of cellular viability as well as the increase of LDH release and caspase-3 activity in ESCC cells.

### 3.2. FA Administration Impairs Colony Formation Capacity and Motivates Apoptosis in ESCC Cells

**Figure 2:**
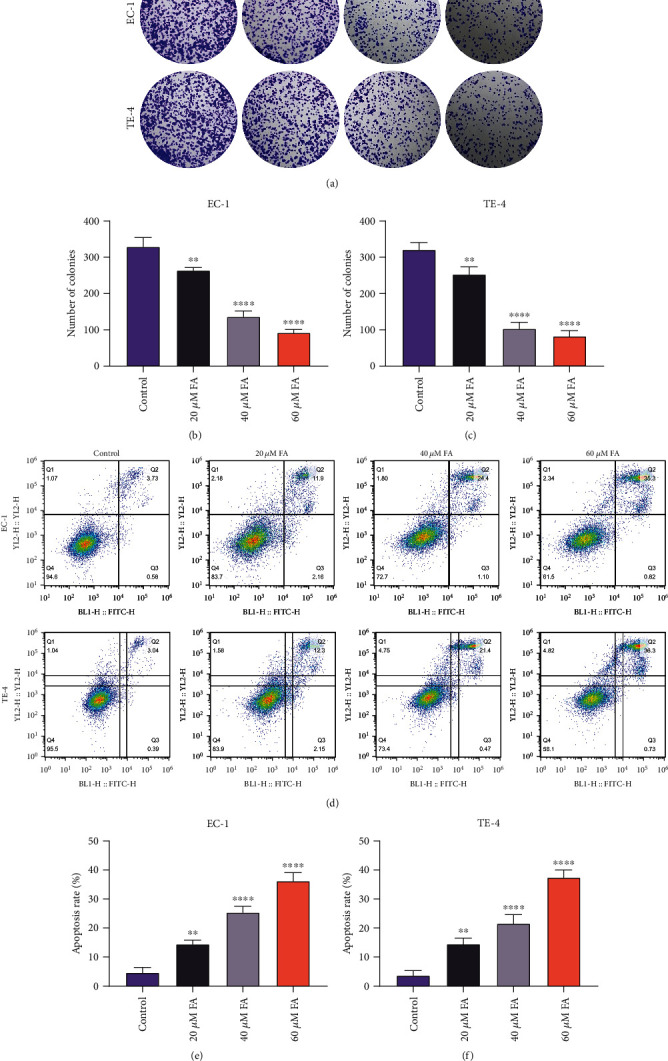
FA administration impairs colony formation capacity and motivates apoptosis in ESCC cells. (a–c) The colonies were counted when EC-1 and TE-4 cells were administrated with 20 *μ*M, 40 *μ*M, and 60 *μ*M FA for 48 h. (d–f) Apoptotic level of EC-1 and TE-4 cells with 48 h administration of 20 *μ*M, 40 *μ*M, and 60 *μ*M FA was tested via Annexin V-FITC apoptosis staining. *p* was computed through one-way ANOVA test. Significance level was denoted as ^∗∗^*p* < 0.01 and ^∗∗∗∗^*p* < 0.0001.

For assessing whether FA exposure resulted in the growth suppression of FA on ESCC cells, the colonies were counted when EC-1 and TE-4 cells were administrated with 20 *μ*M, 40 *μ*M, and 60 *μ*M FA for 48 h. As a result, the data demonstrated that colony formation capacity of ESCC cells was dramatically impaired by gradient doses of FA (Figures 2(a)–2(c)). In addition, apoptotic level was tested through Annexin V-FITC apoptosis staining. As illustrated in Figures 2(d)–2(f), 48 h administration of 20 *μ*M, 40 *μ*M, and 60 *μ*M FA memorably motivated apoptosis of EC-1 and TE-4 cells. Hence, above findings suggested that FA led to growth suppression of ESCC cells.

### 3.3. FA Exerts a Suppressive Effect on Migration, Invasion, and Angiogenesis in ESCC Cells

**Figure 3:**
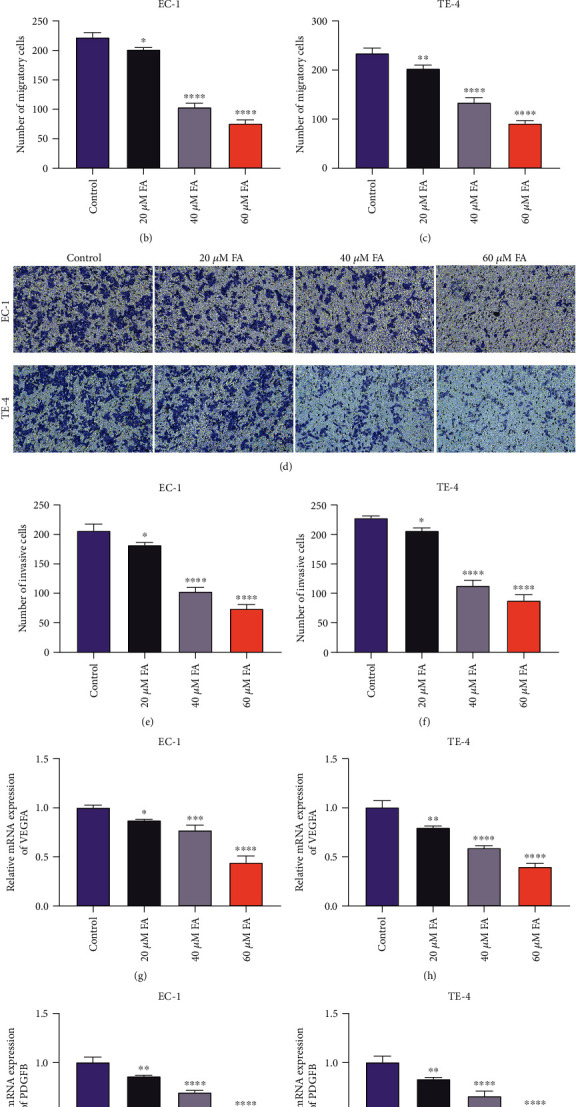
FA exerts a suppressive effect on migration, invasion, and angiogenesis in ESCC cells. (a–c) The migratory cells were counted when EC-1 and TE-4 cells were administrated with 20 *μ*M, 40 *μ*M, and 60 *μ*M FA for 48 h via Transwell experiment. Scale bar, 100 *μ*m. (d–f) The invasive EC-1 and TE-4 cells with 48 h administration of 20 *μ*M, 40 *μ*M, and 60 *μ*M FA were counted through Transwell experiment. Scale bar, 100 *μ*m. (g, h) VEGFA mRNA level was measured in EC-1 and TE-4 cells that were administrated with 20 *μ*M, 40 *μ*M, and 60 *μ*M FA for 48 h utilizing quantitative RT-PCR analysis. (i, j) PDGFB mRNA level was tested in EC-1 and TE-4 cells with 48 h administration of 20 *μ*M, 40 *μ*M, and 60 *μ*M FA through adopting quantitative RT-PCR analysis. *p* was computed through one-way ANOVA test. Significance level was denoted as ^∗^*p* < 0.05, ^∗∗^*p* < 0.01, ^∗∗∗^*p* < 0.001, and ^∗∗∗∗^*p* < 0.0001.

Through adopting Transwell experiments, the current study investigated the influence of FA on migratory and invasive capacities of ESCC cells. As illustrated in Figures 3(a)–3(c), the number of migratory EC-1 and TE-4 cells was observably diminished under 48 h exposure of 20 *μ*M, 40 *μ*M, and 60 *μ*M FA. In addition, FA administration contributed to the reduction of invasive EC-1 and TE-4 cells (Figures 3(d)–3(f)). Hence, FA exerted a suppressive property on migration together with invasion in ESCC cells. Afterwards, the impact of FA on ESCC angiogenesis was assessed through measuring angiogenesis-relevant genes. Consequently, 48 h administration of 20 *μ*M, 40 *μ*M, and 60 *μ*M FA memorably lessened the levels of VEGFA and PDGFB mRNAs in C-1 and TE-4 cells (Figures 3(g)–3(j)), indicating that FA led to the angiogenesis suppression of ESCC cells.

### 3.4. FA Administration Leads to Oxidative Stress Damage of ESCC Cells through Lipid Peroxidation and ROS Generation

**Figure 4:**
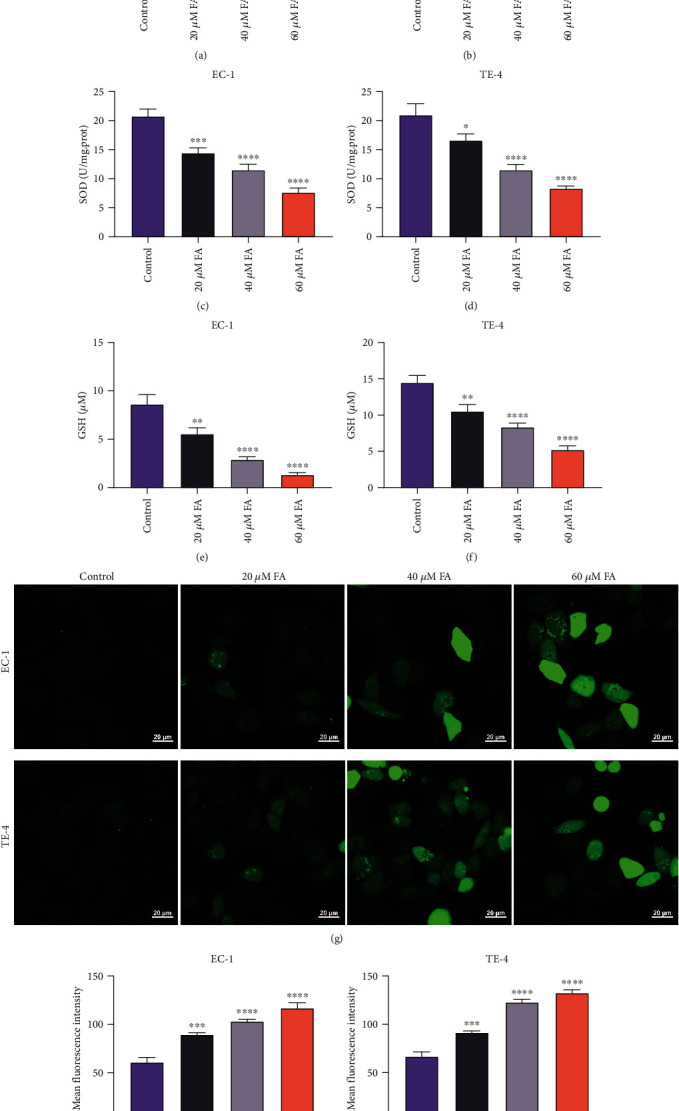
FA administration leads to oxidative stress damage of ESCC cells through lipid peroxidation and ROS generation. (a, b) MDA content was detected in EC-1 and TE-4 cells with 48 h administration of 20 *μ*M, 40 *μ*M, and 60 *μ*M FA through MDA content kit. (c, d) SOD activity was monitored and normalized in ESCC cells that were administrated with 20 *μ*M, 40 *μ*M, and 60 *μ*M FA for 48 h utilizing SOD activity kit. (e, f) GSH content was quantified in EC-1 and TE-4 cells following exposure to 20 *μ*M, 40 *μ*M, and 60 *μ*M FA for 48 h. (g–i) Intracellular ROS accumulation was tested in EC-1 and TE-4 cells with 48 h administration of 20 *μ*M, 40 *μ*M, and 60 *μ*M FA utilizing DCFH-DA probe. Scale bar, 20 *μ*m. *p* was computed through one-way ANOVA test. Significance level was denoted as ^∗^*p* < 0.05, ^∗∗^*p* < 0.01, ^∗∗∗^*p* < 0.001, and ^∗∗∗∗^*p* < 0.0001.

The loss of redox homeostasis results in the pathogenesis of ESCC [23]. One of the consequences of oxidative stress is lipid peroxidation, which is reflected by MDA at the cellular level. Under administrated with 20 *μ*M, 40 *μ*M, and 60 *μ*M FA for 48 h, MDA content was notably elevated in EC-1 and TE-4 cells (Figures 4(a) and 4(b)). SOD and GSH are important members of the antioxidant enzyme system. SOD activity together with GSH content in EC-1 and TE-4 cells were observably lessened by FA treatment (Figures 4(c)–4(f)). Afterwards, intracellular ROS generation was monitored in EC-1 and TE-4 cells utilizing DCFH-DA probe. Consequently, 48 h administration of 20 *μ*M, 40 *μ*M, and 60 *μ*M FA dramatically motivated ROS generation in ESCC cells (Figures 4(g)–4(i)). Hence, FA enabled to result in oxidative stress damage of ESCC cells via lipid peroxidation and ROS production.

### 3.5. FA Exposure Contributes to Ferroptotic Cell Death of ESCC Cells

**Figure 5:**
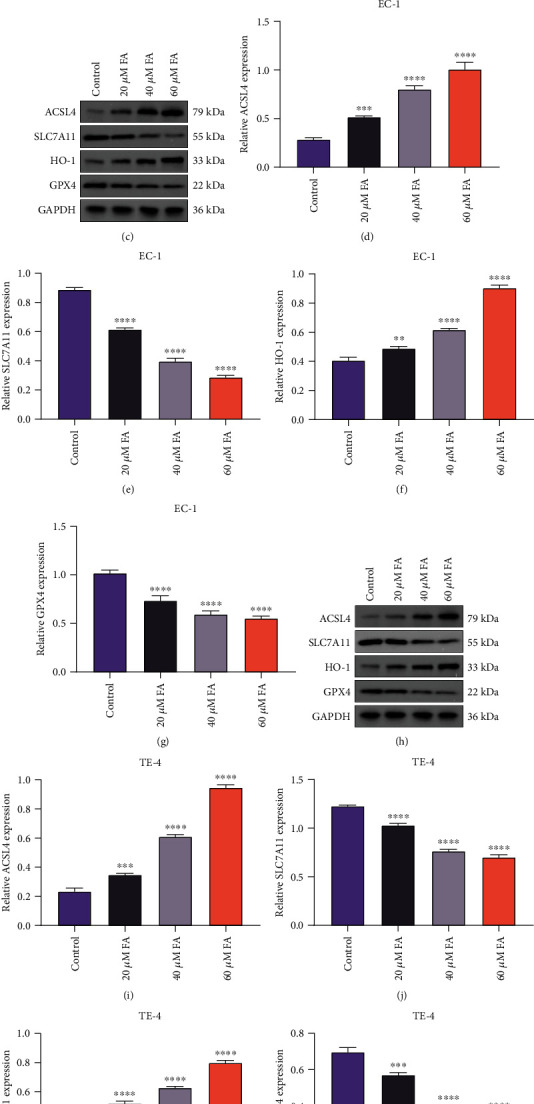
FA exposure contributes to ferroptotic cell death of ESCC cells. (a, b) Iron content was detected in EC-1 and TE-4 cells with 48 h administration of 20 *μ*M, 40 *μ*M, and 60 *μ*M FA through iron content kit. (c–g) Activities of ACSL4, SLC7A11, HO-1, and GPX4 were measured in EC-1 cells following exposure to 20 *μ*M, 40 *μ*M, and 60 *μ*M FA for 48 h through immunoblotting. (h–l) Activities of ACSL4, SLC7A11, HO-1, and GPX4 were tested in TE-4 cells that were administrated with 20 *μ*M, 40 *μ*M, and 60 *μ*M FA for 48 h via adopting immunoblotting. *p* was computed through one-way ANOVA test. Significance level was denoted as ^∗^*p* < 0.05, ^∗∗^*p* < 0.01, ^∗∗∗^*p* < 0.001, and ^∗∗∗∗^*p* < 0.0001.

Further analysis was conducted for assessing whether FA impacted ferroptosis of ESCC cells. We measured iron content in ESCC cells with 48 h administration of 20 *μ*M, 40 *μ*M, and 60 *μ*M FA. The data showed that FA notably increased iron content in EC-1 and TE-4 cells in a dose-dependent manner (Figures 5(a) and 5(b)). In addition, ferroptosis-relevant markers (ACSL4, SLC7A11, HO-1, and GPX4) were determined via immunoblotting. Both in EC-1 and TE-4 cells, FA exposure dramatically augmented the activities of ACSL4 and HO-1 as well as lessened the activities of SLC7A11 and GPX4 (Figures 5(c)–5(l)). These data demonstrated that FA exposure resulted in ferroptotic cell death of ESCC cells.

### 3.6. DFO Impairs the Stimulative Effect of FA on Lipid Peroxidation and ROS Generation in ESCC Cells

**Figure 6:**
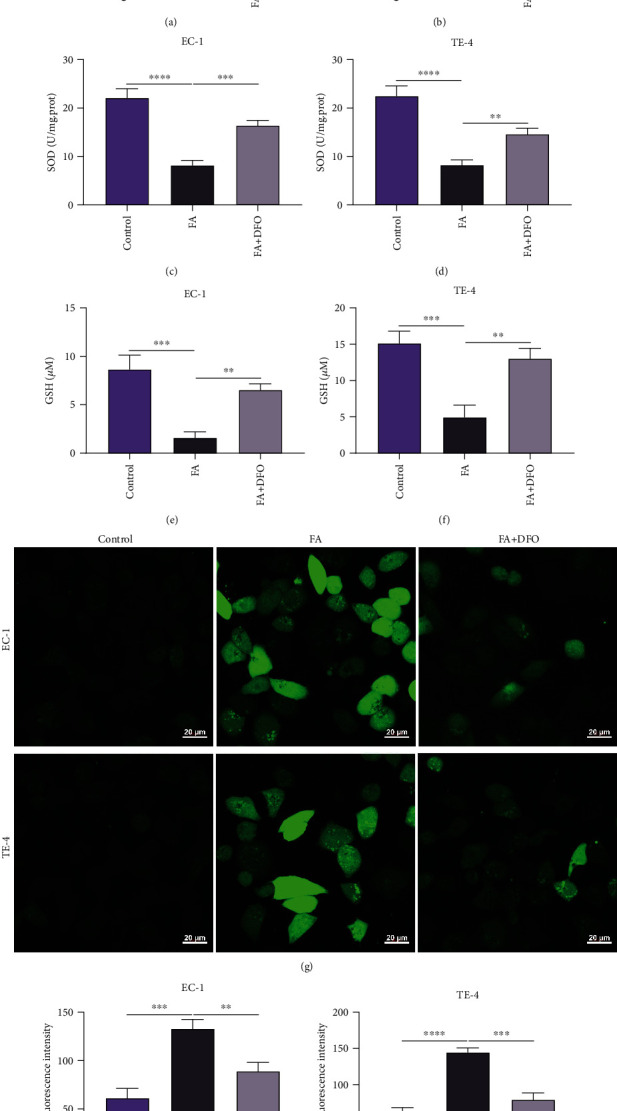
DFO impairs the stimulative effect of FA on lipid peroxidation and ROS generation in ESCC cells. (a, b) MDA content was measured in EC-1 and TE-4 cells with 48 h administration of 40 *μ*M FA and 50 *μ*M DFO by MDA content kit. (c, d) SOD activity was tested in EC-1 and TE-4 cells that were administrated with 40 *μ*M FA and 50 *μ*M DFO for 48 h through adopting SOD activity kit. (e, f) GSH content was assayed in EC-1 and TE-4 cells under exposure to 40 *μ*M FA and 50 *μ*M DFO for 48 h. (g–i) Intracellular ROS accumulation was assessed in EC-1 and TE-4 cells with 48 h administration of 40 *μ*M FA and 50 *μ*M DFO via DCFH-DA probe. Scale bar, 20 *μ*m. *p* was computed through one-way ANOVA test. Significance level was denoted as ^∗∗^*p* < 0.01, ^∗∗∗^*p* < 0.001, and ^∗∗∗∗^*p* < 0.0001.

Ferroptosis is an iron-dependent cell death correlated to peroxidation of lipids. EC-1 and TE-4 cells were coadministrated with 40 *μ*M FA and 50 *μ*M ferroptosis inhibitor DFO for 48 h to observe whether DFO hindered the stimulative effect of FA on lipid peroxidation and ROS production. The data showed that DFO notably whittled the stimulative effect of FA on MDA content together with the suppressive effect on SOD activity and GSH content in EC-1 and TE-4 cells (Figures 6(a)–6(f)). In addition, FA-induced ROS generation was observably weakened by DFO in ESCC cells (Figures 6(g)–6(i)). Hence, DFO enabled to impair the stimulative effect of FA on lipid peroxidation together with intracellular ROS accumulation in ESCC cells.

### 3.7. DFO Restrains the Stimulative Effect of FA on Ferroptotic Cell Death of ESCC Cells

**Figure 7:**
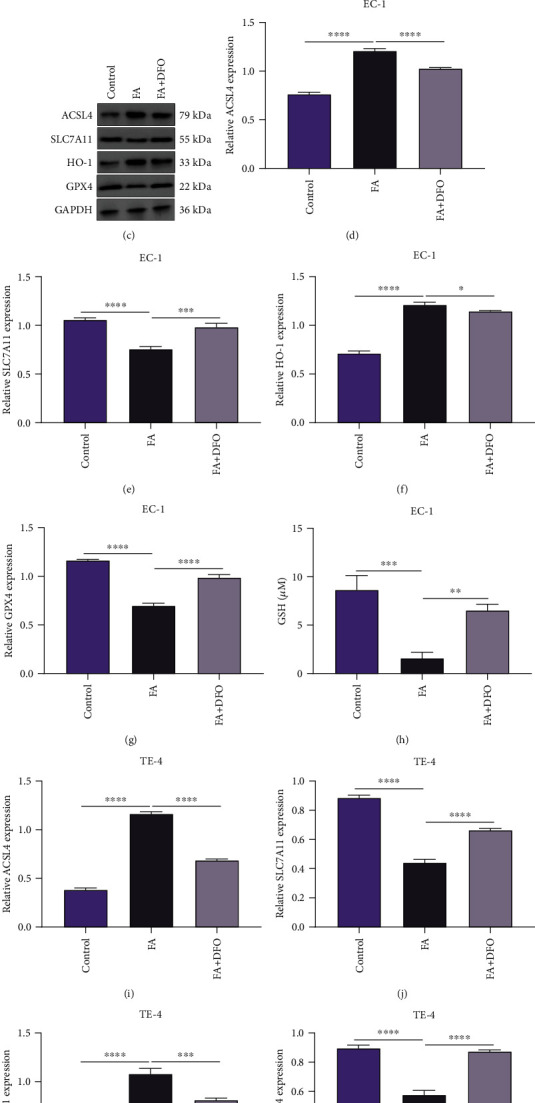
DFO restrains the stimulative effect of FA on ferroptotic cell death of ESCC cells. (a, b) Iron content was measured in EC-1 and TE-4 cells with 48 h administration of 40 *μ*M FA and 50 *μ*M DFO. (c–g) Activities of ACSL4, SLC7A11, HO-1, and GPX4 were tested in EC-1 cells with administration of 40 *μ*M FA and 50 *μ*M DFO for 48 h via immunoblotting. (h–l) Activities of ACSL4, SLC7A11, HO-1, and GPX4 were monitored in TE-4 cells that were exposed to 40 *μ*M FA and 50 *μ*M DFO for 48 h via immunoblotting. *p* was computed through one-way ANOVA test. Significance level was denoted as ^∗^*p* < 0.05, ^∗∗^*p* < 0.01, ^∗∗∗^*p* < 0.001, and ^∗∗∗∗^*p* < 0.0001.

The current study conducted in-depth analysis to assess the property of FA on inducing ESCC ferroptosis. The data showed that DFO markedly lessened the iron content of FA-administrated EC-1 and TE-4 cells (Figures 7(a) and 7(b)). Administration with FA observably heightened the activities of ACSL4 and HO-1 and lessened the activities of SLC7A11 and GPX4 in EC-1 and TE-4 cells, which was reversed by DFO co-administration (Figures 7(c)–7(l)). Hence, DFO restrained the stimulative effect of FA on ferroptotic cell death of ESCC cells.

## 4. Discussion

Ferroptosis is an iron- and lipid ROS-dependent cell death type, which morphologically, biologically, and genetically differentiates from other cell death types [24]. Evidence demonstrates the crucial functions of ferroptotic cell death against ESCC [25]. Ferroptosis is mitigated in ESCC and acts as a dynamic tumor suppressor in ESCC progression, suggesting that inducing ferroptosis may be applied as a potential interventional target for ESCC therapy [16]. Hence, small molecules reprogramming ESCC cells to experience ferroptotic cell death are regarded as potent agents for treating ESCC.

The potency of FA in cancer therapeutics has been proposed in other cancer types. For instance, FA attenuates proliferation and induces apoptosis through blocking PI3K/Akt signaling in osteosarcoma [26]. In addition, it mitigates cellular viability together with colony formation in pancreatic cancer [27]. Nonetheless, whether FA mitigated ESCC progression has not been reported. The current experiments demonstrated that FA attenuated cellular viability and colony formation capacity and motivated LDH release, caspase-3 activity, and apoptosis in EC-1 and TE-4 cells. Thus, ESCC cells might be vulnerable to FA. In addition, we observed the suppressive effect of FA on migration and invasion together with angiogenesis through VEGFA and PDGFB in ESCC cells, consistent with previously published literature [28, 29].

Ferroptosis is initiated by redox imbalance between the generation of oxidants and antioxidants, triggered by the aberrant expression and activities of various redox-active enzymes generating free radicals together with lipid oxidant products [30]. This cell death form has the features of elevated levels of lipid hydroperoxides and iron overload, resulting in caspase- and necrosome-independent cell death [31]. FA administration resulted in the increase of MDA content, ROS production, and iron load as well as the reduction of SOD activity and GSH content. ACSL4 may dictate the sensitivity ferroptotic cell death through shaping cellular lipid compositions [32]. Also, phosphorylation of ACSL4 by PKC*β*II amplifies lipid peroxidation to trigger ferroptosis [33]. Inducing ferroptosis enables to attenuate lung cancer cell growth together with migration [34]. HO-1 is recognized as a survival indicator of cancer cells as well as a ferroptosis inducing molecule [35]. The cystine/glutamate antiporter SLC7A11 is utilized for importing cystine for glutathione biosynthesis as well as antioxidant defense [36]. Recently, SLC7A11 mediated by NRF2 enhances ESCC radiosensitivity via attenuating ferroptotic cell death [14]. In addition, SLC7A11 functions as an independent prognostic indicator in human ESCC [37]. Selenium-containing enzyme GPX4 is regarded as a central inhibitor of ferroptotic cell death, and its activity depends upon glutathione generated from SLC7A11 activation [38]. In ESCC cells, FA augmented the activities of ACSL4 and HO-1, with cutting down SLC7A11 and GPX4. In addition, DFO restrained the effect of FA on ESCC ferroptosis. Altogether, FA mitigated growth together with invasion of ESCC through inducing ferroptotic cell death.

## 5. Conclusion

Thus, the current study is aimed at investigating the functional property of FA on inducing ferroptotic cell death in anti-ESCC. Our findings unveiled FA as a novel ferroptosis inducer, thus attenuating cell growth and invasion of ESCC, which might boost the clinical application of FA in ESCC therapeutics.

## Data Availability

The datasets analyzed during the current study are available from the corresponding author on reasonable request.

## Abbreviations

ESCC: Esophageal squamous cell carcinoma
ROS: Reactive oxygen species
FA: Ferulic acid
DMSO: Dimethyl sulfoxide
DFO: Deferoxamine
CCK-8: Cell Counting Kit-8
LDH: Lactate dehydrogenase
RT: Room temperature
SFM: Serum-free medium
MDA: Malondialdehyde
SOD: Superoxide dismutase
GSH: Glutathione
DCFH-DA: Dichlorodihydrofluorescein diacetate.
